# Chemopreventive Role of Black Tea Extract in Swiss Albino Mice Exposed to Inorganic Arsenic

**DOI:** 10.31557/APJCP.2021.22.11.3647

**Published:** 2021-11

**Authors:** Archismaan Ghosh, Sutapa Mukherjee, Madhumita Roy

**Affiliations:** *Department of Environmental Carcinogenesis and Toxicology Chittaranjan National Cancer Institute 37, S P Mukherjee Road, Kolkata, India. *

**Keywords:** Inorganic arsenic- skin carcinogenesis- black tea extract- ROS

## Abstract

**Objective:**

Chronic exposure to inorganic arsenic (iAs) may cause a number of health problems including skin cancer. Present study is aimed to look into the potential of black tea extract (BTE) to prevent the development of skin carcinoma in Swiss albino mice.

**Methods:**

The study was done on Swiss albino mice, chronically exposed to inorganic arsenic. 150 mice were housed in different cages, 5 in each cage. The control mice did not receive any treatment. Mice were sacrificed at 30, 90, 180, 270 and 330 days. Development of carcinogenesis was assessed by histological studies. Generation of Reactive Oxygen Species (ROS) and Reactive Oxygen Species (RNS) were estimated using 2’,7’-dichlorodihydrofluorescein diacetate (DCFH-DA) and Greiss reagent respectively, and their consequences on DNA (by Micronuclei and Comet assay), protein (by protein carbonyl assay kit) and lipid (by lipid peroxidation) were estimated. Activity of antioxidant enzymes, along with total antioxidant capacity were measured by respective kits. Repair percentage was obtained by Comet assay. Western blotting was employed to study the expression of repair enzymes and expression of cytokines. Sandwich Enzyme-linked Immunosorbent Assay (ELISA) technique was employed to study the activity of various cytokines.

**Results:**

At 330 days, invasive squamous cell carcinoma of the skin developed. With increasing time generation of ROS and RNS increased, causing damage to DNA, protein and lipid. Antioxidant defence system gets repressed with time. Capacity to repair the DNA damage is inhibited by iAs, due to alteration in repair enzymes - XRCC I, DNA Ligase I, PARP I, ERCC1, ERCC2, XPA, DNA Ligase IV, DNA PKc and Ku-70. Another consequence of iAs exposure is chronic inflammation due to disrupted cytokine level. Intervention with BTE reverses these deleterious effects, preventing development of skin carcinogenesis.

## Introduction

Chronic exposure to the metalloid arsenic, which exists in both organic and inorganic forms, may cause numerous health hazards (Tchounwou et al., 2012), and the inorganic form is more toxic (Hughes et al., 2011). Chronic exposure to inorganic arsenic (iAs) may result in a plethora of diseases, both non-malignant, as well as malignant (Martinez et al., 2011) and cancer of skin, kidney, bladder, lung etc. are endemic. 

Excess generation of reactive oxygen species (ROS) due to iAs is an important contributory factor to its carcinogenicity (Zhang et al., 2015). Higher level of ROS mediates its damage at the cellular level, affecting nucleic acids, lipids and proteins (Pizzino et al., 2017). Higher fluxes of ROS generation lead to formation of protein carbonyl due to oxidation of amino acid residues (Sinha et al., 2010). High level of ROS also leads to lipid peroxidation of polyunsaturated fatty acids, generating malondialdehyde, which elicits cytotoxic effects and damage to cell membrane. Defragmentation of lipid chains, resulting in excess fluidity and enhanced permeability of the cell membrane may occur due to ROS (Yusupov et al., 2010). Antioxidants are important for reducing damage due to ROS.

Antioxidant enzymes act in defence of the cells against excess ROS induced DNA, lipid and protein damage, maintaining homeostasis by reducing the damage. Some of these antioxidant enzymes are superoxide dismutase (SOD), catalase, Glutathione peroxidase (GPX), Glutathione reductase (GR), Glutathione transferase (GT) etc. (Caverzan et al., 2016). Normally a balance between ROS and antioxidant defence is maintained, preventing permanent damage, either by removal or by efficient repair of the damaged parts. There are certain repair mechanisms: (i) base excision repair (BER), which repairs single-strand breaks due to oxidative DNA damage; (ii) nucleotide excision repair (NER) that repairs DNA cross links and bulky adducts due to insult by environmental challenges; (iii) nonhomologous end joining (NHEJ) which is implicated in DNA repair in eukaryotic systems (Rowe et al., 2008). All these act to counter DNA damage. Chronic exposure to iAs leads to impairment of repair mechanisms due to inhibition of a number of repair enzymes, which is another contributing factor for carcinogenesis (Sinha and Roy, 2011). Exposure to iAs has been found to weaken the activities of several antioxidant enzymes. Inflammation is the cornerstone of many diseases. ROS is also known to stimulate the activation of transcription factor NF-κB (nuclear factor kappa-light-chain-enhancer of activated B cells) via phosphorylation and inhibition of IκBα (nuclear factor of kappa light polypeptide gene enhancer in B-cells inhibitor, alpha) (Maru et al., 2014). Phosphorylation of the tyrosine residues of IκBα by ROS leads to its degradation, thereby activating NF-κB. NF-κB is the downstream effector molecule of inflammatory response and modulates many pathways leading to cell growth and proliferation (Hoesel and Schmid, 2013).

Exposure to a number of pollutants including arsenic can trigger extrinsic inflammatory response, whereas mutations and oncogenic activation via recruitment of inflammatory cells can result in onset of intrinsic response (Maru et al., 2014). Substances triggering extrinsic pathway may lead to tissue injury and infiltration of neutrophils in skin. This further leads to production of ROS and reactive nitrogen species (RNS), and finally results in alteration of metabolism, cell cycle and other intercellular pathways (Reuter et al., 2010). These reactive species play an important role in all stages of carcinogenesis namely initiation, promotion and progression (Ibanez et al., 2011). Inflammation, which is listed as one of the hallmarks of cancer (Hanahan and Weinberg, 2011) can be acute or chronic, of which the latter promotes tumorigenesis. Both the inflammatory pathways contribute to tumorigenesis via immunosuppression. Immune response induces the secretion of molecules like cytokines, chemokines as well as growth factors which are paracrine and autocrine in function. 

Cytokines play a pivotal role in controlling inflammatory environment and are responsible for both anti and pro-tumorigenic roles. Of these, pro-inflammatory cytokines aid in chronic inflammation and thus have prominent role in development of skin cancer. Pro-inflammatory cytokines like TGF-β (transforming growth factor beta), TNF-α (tumour necrosis factor alpha), interkeukins (IL) like IL-1β, and IL-6 (Interleukin) elevate the expression of selectins on the vascular cells, which synchronize with the IL-8 secreted by the tumor cells (Mantovani et al., 2008). 

Inflammation plays a lead role in skin cancer, in which Basal Cell carcinoma (BCC) and Squamous cell carcinoma (SCC) are common (Avrămoiu et al., 2016). Inflammatory markers can be used in diagnosis for skin cancer, e.g IL-17 and IL-22, which induce cell proliferation and migration in both SCC and BCC. IL-17 along with TNF-α promotes the expressions of IL-6 and IL-8. In animal models IL-17 and IL-22 play important roles in tumorigenesis (Nardinocchi et al., 2015). TNF-α plays a dual role i.e., it may exhibit pro, as well as anti-tumor activity. Death of cancer cells is facilitated due to its anti-tumorigenic activity, while as a pro-tumorigenic factor it activates NF-κB, promoting cell survival, cell proliferation and ultimately cancer. 

Quenching of ROS may be a viable strategy to counter iAs induced oxidative stress. Promising antioxidant N-acetyl cysteine (NAC), a precursor of L-cysteine, is a direct scavenger of free radicals, due to its sulfhydryl group (–SH) (Šalamon et al., 2019). Apparently, NAC is a safe molecule, but may have certain toxicities at higher doses (Deepmala et al., 2015). Therefore, non-toxic plant derived molecules called polyphenols with strong antioxidant activity may offer respite from these side effects. 

Being good antioxidants, polyphenols downregulate the catalytic activity of the enzymes triggering ROS production, quenching ROS and upregulating antioxidant activity, due to their low redox potential (Mishra et al., 2013). Flavonoids, a class of polyphenols can attach to the non-polar, hydrophobic, cytosolic end of the plasma membrane and prevent the oxidation and damage of the cell membrane (Oteiza et al., 2005). Among other attributes, these active biomolecules may interact with nitric oxide synthetase (NOS) altering nitric oxide production (Sarkar and Bhaduri, 2001). These active molecules modulate inflammatory pathways and alter the secretion of pro-inflammatory molecules (Yahfoufi et al., 2018). Tea, the most favoured beverage around the globe is rich in antioxidants like flavonoids, by virtue of which it aids in prevention of cell damage, rejuvenation of antioxidant enzymes, reduction of inflammation etc. (Chatterjee et al., 2012; Sen and Bera, 2013). All these events assist to fight against cancer. 

Green tea is already known for its antioxidant potential, anti-tumorigenic effect and it acts on all stages of carcinogenesis. Black tea (BT), on the other hand is more popular. Reports from our laboratory showed that polyphenols in BT are no less potent than those found in green tea (Sinha et al., 2003). It was found that Theaflavins in BT may counter the cytotoxicity of arsenic and are comparable to Catechins found in green tea (Sinha et al., 2003). 

Hence, it is worthwhile to explore the potential of back tea extract against arsenic induced skin cancer. The aim of this study is to evaluate the effect of black tea extract on prevention of skin cancer in Swiss Albino mice exposed to inorganic arsenic.

## Materials and Methods

*Animal maintenance*

4-5 weeks old male Swiss Albino (Mus musculus) mice were taken. Altogether 150 mice were taken, 5 in each group. Ethical permission was granted by the institutional animal ethical committee (IAEC 1774/MR-3/2017/9). Mice were fed synthetic pellets and water *ad libitum*; maintained with a constant temperature of 22±2°C at 12 hours of darkness and light. The mice were sacrificed by euthanasia using sodium thiopentone over-dose (100 mg/kg body wt). 

*Animal treatment*

The mice were divided into three groups - control, DMBA (7,12-Dimethylbenz[a]anthracene) and iAs. Half of the mice in each group received BTE. Mice were sacrificed at different time points. Detailed doses and treatment protocols are given in Table 1. 

Blood and tissues were collected from sacrificed mice to carry out the experiments. 

*Histology*

Skin tissues fixed in 10% neutral buffered formalin (NBF) were dehydrated with alcohol, cleared with xylene, embedded in paraffin and cut into 4 μm thin sections. They were stained with Haematoxylin and Eosin, mounted with DPX and observed under light microscope. 

*Intracellular ROS estimation*

Leukocytes were separated from the blood of mice using solution A (NH_4_Cl in TRIS at pH 7.2) and solution B (meso-inositol), then suspended in HBS (Hepes Buffered Saline pH 7.4). ROS was estimated following the protocol of Balasubramanyam (2003) with slight modifications (Sinha et al., 2010). Fluorescent intensity was recorded using a Varian Cary Eclipse spectrofluorimeter after staining with 10 mM of 2’,7’ dichlorodihydro-fluorescein (DCFH-DA) for 45 min in the dark. The excitation and emission ranges were 485 nm and 530 nm respectively.

*Intracellular RNS estimation*

RNS generation was measured in the isolated leukocytes using Griess reagent (1% sulphanilamide, 0.1% naphthylethylenediamine-dihydrochloride and 5% orthophosphoric acid) (Green et al., 1982). Absorbance was measured at 550 nm using a spectrophotometer after incubation with Griess’s reagent at 26°C for 30 minutes in a humidified chamber.

*Determination of micronuclei from the bone marrow tissues*

In vivo micronuclei assay was performed by administering three doses (2 mg/kg bw) of cytochalasin-B (to obtain binucleate cells), at an interval of 36 hrs (Umadevi et al., 1997). Bone marrow cells were collected by flushing the femur of the mice with normal saline. Cells, fixed in a fixative (methanol:glacial acetic acid::3:1), were spread on chilled glass slides. They were then stained with 5 % Giemsa and observed under light microscope and calculated as number of micronuclei per 1,000 binucleate cells.

*Single cell gel electrophoresis assay*

Single Cell Gel electrophoresis (SCGE) or comet assay was performed according to Singh (1988), with slight modifications (Sinha et al, 2010). The isolated leukocytes, suspended in 0.6% low melting agarose were spread over frosted slides, pre-coated with normal melting agarose (0.75%). The cells were lysed overnight at 4°C in a lysis buffer, followed by electrophoresis for 20 minutes. Ethidium-bromide stained cells were observed under fluorescent microscope (Leica). The image analysis software Komet 5.5 was used to assess the DNA damage by measuring the comet tail moment and tail length.

*Estimation of Lipid peroxidation assay*

Lipid peroxidation (LPO) was performed according to Okhawa (1979), with slight modifications. To the homogenised tissues, 10% SDS, 20% acetic acid, 0.8% thio-barbituric acid (TBA) were added and boiled for 1 hour. It was then ice cooled immediately for 10 minutes, centrifuged at 2,500 rpm for 10 minutes. Absorbance was recorded at 535 nm and LPO was measured as moles of Malondialdehyde (MDA) generated.

*Measurement of Protein carbonyl content*

The protein carbonyl content was assayed according to the protocol of Protein Carbonyl Colorimetric Assay Kit by Cayman Chemicals (10005020). It measures the protein carbonyl content from the tissue homogenates by using 2,4-Dinitrophenylhydrazine (DNPH), which in the presence of protein carbonyl forms a Schiff’s base which can be measured spectrophotometrically at 360-385 nm.

*Measurement of total antioxidant capacity*

Total antioxidant capacity was measured from the serum using Antioxidant Assay Kit (709001) from Cayman Chemicals. The assay determines the antioxidant capacity of the sample by measuring its ability to prevent the oxidation of ABTS [2,2’-Azino-di-(3-ethylbenzthiazoline sulphate)]. Absorbance was measured at 405 nm. Ability of the antioxidants to prevent the oxidation of ABTS was compared with that of a standard Trolox (Tocopherol analogue). 

*Measurement of Catalase activity*

The peroxidatic function of catalase was measured from the homogenized tissues using Catalase Assay Kit (707002) from Cayman Chemicals. Catalase produces formaldehyde in presence of methanol and H_2_O_2_, which is measured by the chromogen 4-amino-3-hydrazino-5-mercapto-1,2,4-triazole (Purpald). Upon reaction with aldehydes, the chromogen changes its color from colorless to purple. The OD is recorded at 540 nm and the activity was determined. 

*Measurement of Superoxide Dismutase activity*

Superoxide Dismutase (SOD) activity has been measured in the tissue homogenates by Superoxide Dismutase Assay Kit (706002) from Cayman chemicals. SOD dismutates the superoxide radical generated by Xanthine Oxidase which is detected by the Tetrazolium salt. The absorbance is measured at 460 nm. Active SOD dismutates larger number of radicals thus reducing the OD. Different concentrations of provided bovine erythrocyte SOD was used to make the standard curve.

*Measurement of Glutathione peroxidase activity*

The activity of Glutathione peroxidase was measured by Glutathione Peroxidase Assay Kit (703102) from Cayman chemicals, following the manufacturer’s protocol. The decrease in absorbance at 340 nm was recorded and is used to calculate the activity of glutathione peroxidase.

*Measurement of Glutathione Reductase activity*

Glutathione Reductase activity assay was done by Glutathione Reductase Assay Kit (703202), from Cayman chemicals, according to the protocol. The absorbance was measured at 340 nm using a plate reader (TECAN-infinite M200).

*Measurement of Glutathione S Transferase activity*

Glutathione-S-Transferase (GST) activity has been measured in the tissue homogenates using Glutathione S-Transferase Assay Kit (703302), from Cayman chemicals. The total activity of GST is measured by the conjugation of glutathione with 1-Chloro-2,4-Dinitrobenzene (CDNB) at 340 nm. 

*Determination of DNA repair potential*

The isolated lymphocytes from the mice blood were treated with MNNG (N-methyl-N’-nitro-N-nitrosoguanidine), a DNA damaging agent, for 1 hour. After removal of MNNG, cells were allowed a repair time of 3 hours in fresh medium, then harvested and DNA damage was estimated by SCGE/Comet assay. The reduction in tail length of DNA in comparison to the unrepaired DNA lymphocytes provided the repair percentage of the lymphocytes. 

*Western blot analysis*

Whole proteins extracted from the animal tissues were quantified using Bradford’s method, electrophoresed in sodium dodecyl sulphate polyacrylamide gels (SDSPAGE) and blotted upon a nitrocellulose membrane, which was incubated with primary antibody at 1:1000 ratio at 4°C overnight with constant shaking. After washing, the membrane was incubated with alkaline phosphatase conjugated secondary antibody at 4°C for 2 hours and stained with BCIP-NBT (5-bromo-4-chloro-3-indolyl phosphate- nitro blue tetrazolium). The western blot bands were scanned and quantified by using IMAGE MASTER TM17 2D Elite Software, version 3.1 (Amersham Pharmacia biotech Ltd., USA).

*Cytokine detection assay*

The TNF-α, IL-2, IL-6, CXCL1(mouse homologue of IL-8), IL-10, IL-13, IL-17A, IL-22 activities were measured in the serum according to the manufacturer’s protocol as mentioned in the Mouse TNF alpha ELISA Kit (ab208348), Mouse IL-2 ELISA Kit (ab223588), Mouse IL-6 ELISA Kit (ab100712), Mouse CXCL1 ELISA Kit (GRO alpha) (ab213859), Mouse IL-10 ELISA Kit (ab255729), Mouse IL-13 ELISA Kit (ab219634), Mouse IL-17A ELISA Kit (ab199081), Mouse IL-22 ELISA Kit (ab223857) by Abcam respectively, utilising the sandwich ELISA technique. The endpoint was measured colorimetrically at 450nm. 

*NF-κB detection assay*

The activity of NF-κB p50 and p65 subunits were assessed by the NF-κB p50 Transcription Factor Assay Kit (ab207217) and NF-κB p65 ELISA Kit (ab176648) respectively. The assay was performed according to the manufacturer’s protocol using the sandwich ELISA technique. The concentration was measured from the OD’s obtained at 450nm absorbance.

*Statistical analysis*

SPSS 10.0 software was used to perform the statistical operations using factorial Analysis of Variance (ANOVA). *p< 0.0001, **p<0.001 and ***p<0.05 were used to consider the significance level in comparison to the control mice, while ^a^p< 0.0001, ^b^p<0.001 and ^c^p<0.05 were used to compare the effect of black tea with the iAs treated mice.

## Results

*Development of Skin carcinogenesis*

Five different groups of mice and corresponding treatment protocols are given in Table 1. 

Histological analysis of the skin tissue of mice, sacrificed at different time of exposure, revealed the development of invasive SCC at 330 days. Hyperplasic changes were first observed in Group II and III mice, after 90 days of treatment, while prominent dysplastic changes were visible 180 days onwards. The mice of group IV and V only showed slight hyperplasic changes even after 180 days of treatment. Development of in situ carcinoma was clearly visible at 270 days of treatment for the mice of groups II and III. Invasive SCC was confirmed in both these groups at 330 days of treatment. On the other hand, no such in situ or invasive carcinoma was observed in the group IV and group V even after 270 and 330 days of treatment, only dysplastic and hyperplasic changes were observed.

*Evaluation of Reactive oxygen species*

**Figure 1:**
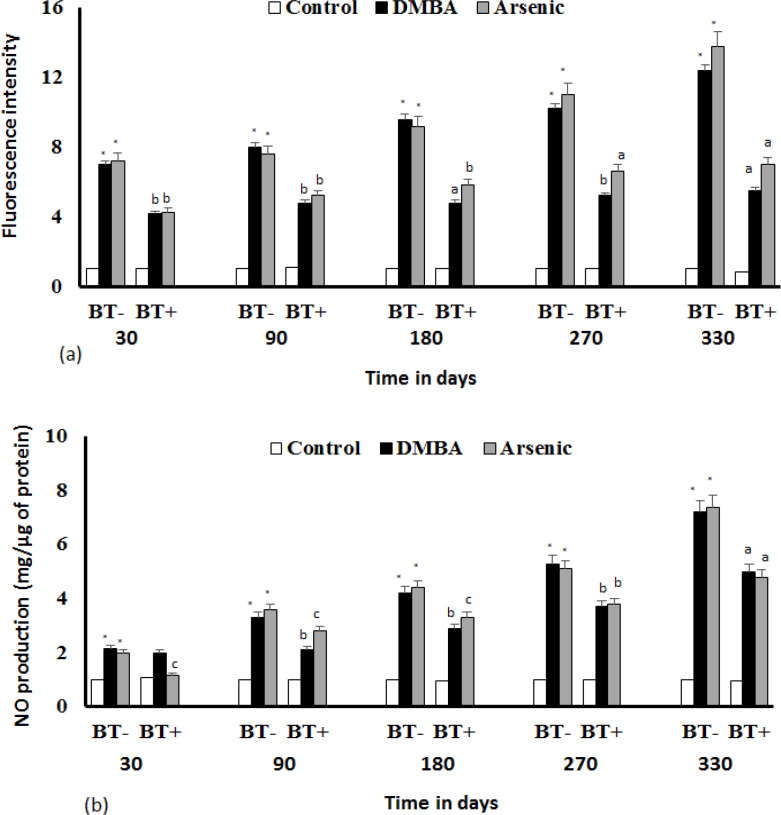
Determination of Free Radical Production in Control, DMBA Treated and iAs Exposed Group and Corresponding BTE Intervention Groups at Different Time Points. (a) Generation of ROS as estimated by spectrofluorimeter after staining with DCF-HDA. (b) Generation of RNS as estimated spectrophotometrically using Greiss reagent. Values are mean of three independent experiments. High level of free radical production generated is significant with respect to control at *p<0.0001. Quenching of free radicals by BTE in comparison to DMBA or iAs treated group is significant at ^a^p<0.0001, ^b^p<0.001, ^c^p<0.05

ROS was estimated at different time intervals (30, 90, 180, 270 and 330 days). The mean of fluorescent intensity of three independent experiments has been represented as a bar graph (Figure 1a). ROS generation, as measured by fluorescent intensity, increased with time in iAs treated mice and DMBA positive control mice. Administration of BTE reduced the ROS level and reduction was appreciably significant (p<0.0001) at 180, 270, 330 days, in comparison to iAs treated group. 

*Evaluation of Reactive nitrogen species*

RNS was estimated using the Griess’ reagent and mean OD was plotted against the time period (Figure 1b). The bar graph clearly indicates that RNS generation was significantly high in iAs and DMBA treated mice (p<0.0001) with increasing time. Significant reduction in generation of RNS was observed in BTE treated groups at 180 days (p<0.001) and 330 days (p<0.0001). 

*Evaluation of DNA damage*

**Figure 2:**
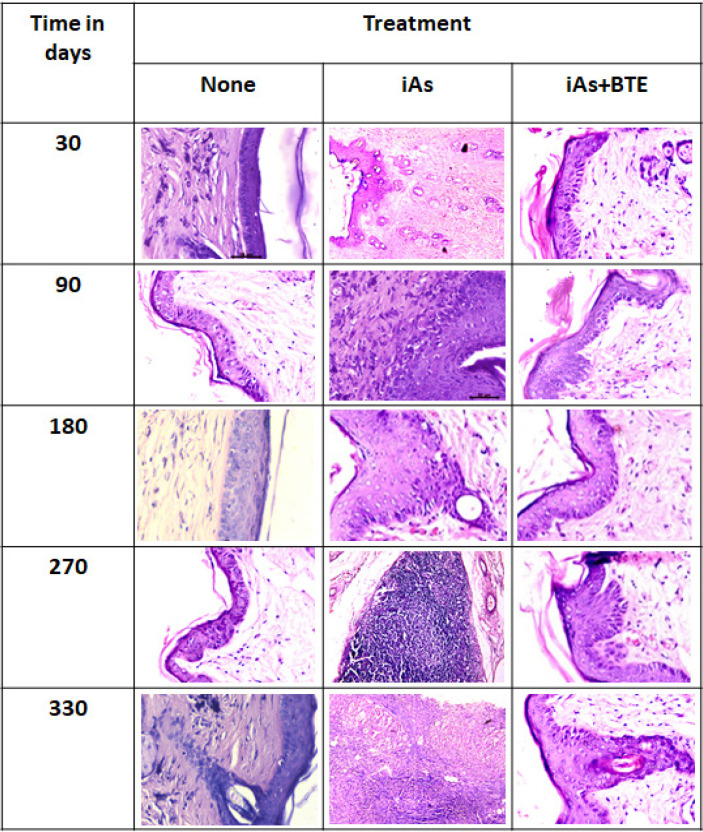
Histopathological Studies during Development of iAs Induced Skin Carcinogenesis at Different Stages. Tissue Sections were Stained by H & E and Visualised under a Light Microscope. Tissue sections indicate hyperplasic changes at 90 days of iAs exposure, which further developed to dysplasia at 180 days, along with appearance of keratin pearls. At 270 days of exposure, in situ carcinogenesis was observed, which further developed into invasive SCC at 330 days. In BTE intervention group, no changes have been observed upto 90 days, beyond which mild hyperplasic changes were observed at 180 days. Low grade dysplastic changes were seen at 270 and 330 days, including formation of keratin pearls. Representative photographs have been taken at 40x magnification

DNA damage was estimated using micronuclei (MN) and Comet assay. The data obtained has been represented as a bar graph representing MN/1000 binucleated cells against time. MN frequency increased with exposure time to DMBA and iAs (Figure 2a), but significantly reduced in BTE administered group (p<0.0001) at 330 days. Comet tail length and Olive tail moment, which are good indices of DNA damage were estimated by SCGE. The broken DNA strands that form a comet pattern due to electrophoresis indicate that the tail length had been increased appreciably (p<0.0001) with exposure time (Figure 2b). The olive tail moment (Figure 2c) is a product of the tail length and % of DNA in the tail, which increases with exposure time, Olive tail moment of the iAs and DMBA group show an increasing trend, which is reduced by inclusion of BTE. At 330 days, both tail length and olive tail moment reduced significantly (p<0.0001). After 330 days of exposure time, class 5 comet pattern was observed, indicating severe DNA damage. 

*Estimation of lipid-peroxidation and protein carbonyl formation*

**Figure 3:**
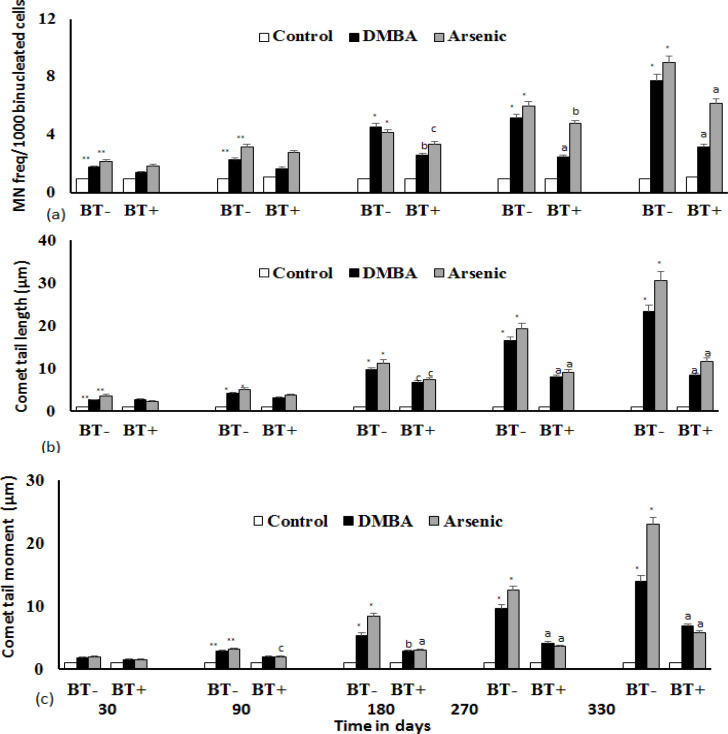
DNA Damage Induced by DMBA and iAs and Its Amelioration by BTE. (a) Prevention of DNA damage as assessed by MN frequency/1000 binucleated cells by BTE, by staining with Giemsa. (b) Prevention of DNA damage by BTE as assessed by Comet tail length. Ethidium bromide stained cells were visualised under a fluorescence microscope after electrophoresis. (c) Prevention of DNA damage by BTE as assessed by Comet tail moment. Data points are mean of three independent experiments. On an average 200 cells were scored by point. Damage induced by DMBA and iAs with respect to control is significantly high. *p< 0.0001; **p<0.001. Reduction of DNA damage by BTE with respect to DMBA or iAs group are significant at ^a^p<0.0001, ^b^p<.001, ^c^p<0.05

Lipid-peroxidation, as measured by moles of malondialdehyde (MDA) generated gives an estimation of lipid damage. The MDA generation increased with exposure time (Figure 3a), indicating that iAs and DMBA showed significant (p<0.0001) lipid damage with progression of cancer from 180 days onwards, which had been found to be reduced by BTE quite appreciably, and at 330 days reduction is highly significant (p<0.0001). Protein damage, as assessed by protein carbonyl formation (Figure 3b) was not much affected up to 90 days, beyond which significant rise in protein damage was observed (p<0.001). Damage in protein was found to be reduced by BTE at 270 days (p<0.001) and 330 days (p<0.0001). Results show a protective role of BTE in lipid and protein damage. 

*Estimation of antioxidant capacity*

**Figure 4:**
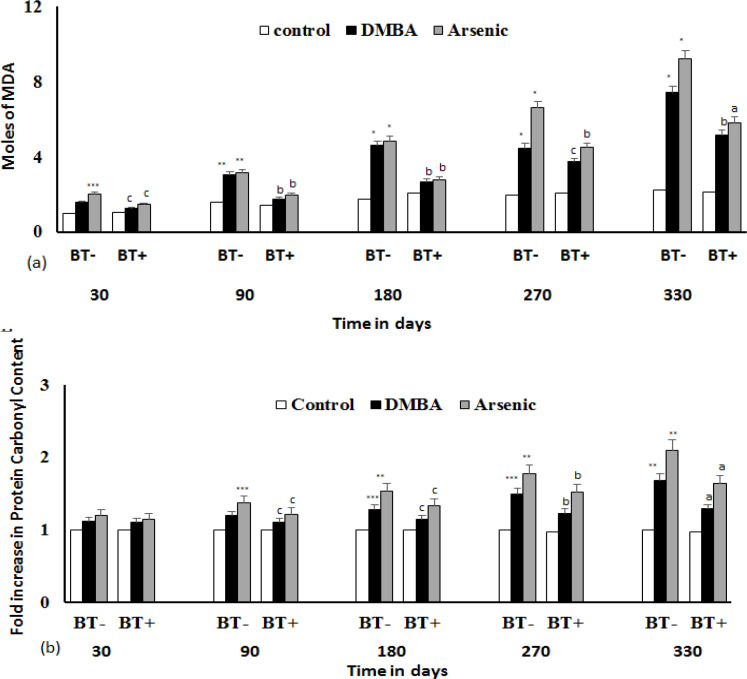
Effect of DMBA and iAs on Lipid and Protein. (a) Prevention of Lipid damage by BTE as assessed by lipid peroxidation assay by calculating the moles of malondialdehyde. (b) Prevention of protein damage by BTE as assessed by formation of protein carbonyl. Damage in lipid and protein induced by DMBA and iAs with respect to control is significantly high. *p< 0.0001; **p<0.001; ***p<0.05. Reduction of lipid and protein damage by BTE with respect to DMBA or iAs group are significant at ^a^p<0.0001, ^b^p<.001, ^c^p<0.05

Estimation of total antioxidant capacity and level of SOD, catalase, GPx, GR and GST have been depicted (Figure 4). With increasing exposure time, total antioxidant capacity (Figure 4a) has been reduced significantly. Significant reduction (p<0.0001) in Trolox equivalent and in enzyme activities with exposure time were observed in Catalase, SOD, GPX and GST (Figures 4b, c, d, f) from 180 days onwards, which were appreciably reversed by inclusion of BTE at 330 days (p<0.0001). GR also showed similar trend, but depletion was much less (Figure 4e). Effect of BTE on GR was not much significant (p<0.05). BTE, which is an antioxidant showed its efficacy against the antioxidant system. 

*Determination of DNA repair capacity and expression of repair enzymes*

**Figure 5:**
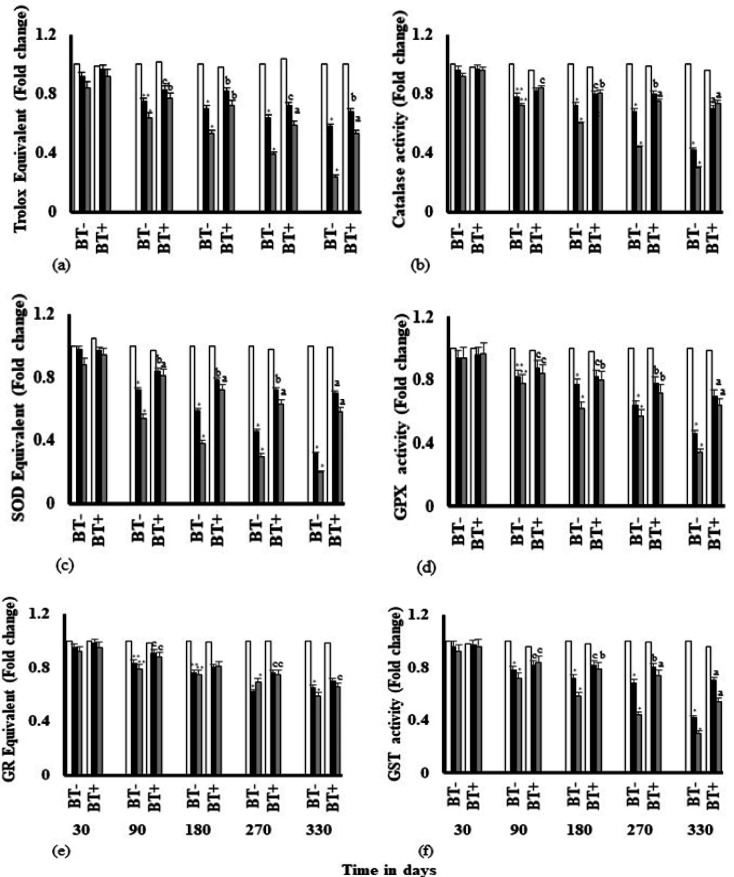
Antioxidant Capacity and Enzymes as Affected by DMBA and iAs. (a) Total antioxidant capacity was determined by Trolox antioxidant assay. Trolox antioxidant activity was expressed as fold change and is the mean of three independent experiments. The bar diagrams in (b), (c), (d), (e), (f) show the fold change in activities of Catalase, SOD, GPX, GR and GST respectively. Data are shown as mean ± SD of three independent experiments. Reduction in Antioxidant capacity and levels of antioxidant enzymes have been found to be reduced significantly with respect to the control, at *p<0.0001; **p<0.001; ***p<0.05. Intervention of BTE have been found to elevate the levels with respect to DMBA and iAs treated groups, significantly at ^a^p<0.0001, ^b^p<.001, ^c^p<0.05

DNA repair capacity estimated in the lymphocytes of mice showed that with increasing exposure time, the repair potential in the iAs treated mice reduced and was significantly low at 330 days (Figure 5a). Repair capacity (RC), expressed as repair %, was studied in lymphocytes after treatment with MNNG for 1 hour to create a damage, followed by a repair time of 3 hours. RC was estimated with respect to the control lymphocytes, which were challenged by MNNG and immediately processed for DNA damage assay, without giving any time to repair; the damage was considered 100%. Results show that the RC of the iAs treated mice was significantly (p<0.0001) reduced after 90 days onwards and BTE reversed the process significantly (p<0.0001). The expressions of repair enzymes X-ray repair cross-complementing protein 1 (XRCC I), DNA Ligase I and Poly (ADP-ribose) polymerase (PARP I) belonging to the BER pathway, excision repair cross complementation (ERCC1, ERCC2) and Xeroderma pigmentosum complementation group A (XPA) belonging to the NER pathway and DNA Ligase IV, DNA-dependent protein kinase (DNA PKc) and Ku-70 (also known as XRCC6) belonging to the NHEJ pathway were investigated. Western blot results revealed decreased expression of the repair enzymes in the iAs treated group with advancement of carcinogenesis, but administration of BTE upregulated the repair enzymes (Table 2). For ERCC1 and DNAPKcs repression of enzymes was significant at the level p<0.001, but for all others reduction was highly significant at the level p<0.0001. Inclusion of BTE enhanced the expression significantly (p<0.0001), except for DNAPKcs. The expression of OGG1 (8-Oxoguanine DNA Glycosylase), involved in repair of 8 oxoguanine (8 oxoG), was found to be upregulated with exposure time in the iAs group (p<0.0001) and downregulated in the BTE administered (p<0.0001) group. β-actin was used as the loading control.

*Estimation of the activity and expression of pro and anti-inflammatory cytokines*

Effect of iAs on the activity of the pro-inflammatory cytokines TNF-α, IL 2, IL6, IL8, IL13, IL17a and IL22 showed that with increasing exposure time, as carcinogenesis advanced, the level increased significantly (p<0.0001) at 330 days. TNF-α and IL22 showed a much higher increase than the other cytokines. Administration of BTE showed reduction in the activity of IL2 and IL6 (p<0.001), BTE has been found to decrease the levels significantly (p<0.0001) at 330 days. A similar trend was also observed in the western blot results where the expressions of pro-inflammatory cytokines were significantly higher when exposed to iAs, but, BTE brought down the elevated expression level. Present findings give an indication that a prolonged inflammatory condition is maintained due to iAs exposure, as evident from the expression and activity of the pro-inflammatory cytokines. On the contrary, the activity and expression of anti-inflammatory cytokine IL10 was seen to be gradually reduced with exposure time in the iAs group; at 330 days reduction was highly significant (p<0.0001). which was prevented by BTE group (p<0.001). These results give a hint about the anti-inflammatory role of the BTE, which showed a promising role in iAs induced inflammation control. 

*Estimation of the activity and expression of NF-κB*

Modulation of NF-κB under the influence of iAs is important, as it plays an important role in regulation of cytokines. The activity and expression of NF-κB subunits p50 and p65 showed significant (p<0.0001) increases by iAs, with exposure time, which was reduced by BTE (p<0.0001) at 180 and 330 days. The findings show similar trends with the pro-inflammatory cytokines. NF-κB, a transcription factor, plays an important role in maintaining cellular homeostasis and is implicated as the downstream regulator of inflammation. Present study indicates that BTE maintains normal homeostasis by regulation of NF-κB. 

**Table 1:** Treatment of Different Mice Groups

Group	"DMBA 100 μg/100μl, painted on the shaved hind part, one shot + Croton oil as promoter"	"iA 500 µg/l, given as the only source of water + painted on the shaved hind part, once daily"	"BTE 0.33 mg/gm body weight, at an interval of 8 hours by oral gavage "
I	-	-	-
II	+	-	-
III	-	+	-
IV	+	-	+
V	-	+	+

150 mice were housed in different cages, 5 in each cage. Mice were sacrificed at 30, 90, 180, 270 and 330 days. Group I, not receiving DMBA, iAs or BTE is the control group.

**Table 2:** Fold Changes in the Expression of Repair Enzymes by iAs as Evident from Band Intensities and Their Modulation by BTE

Repair Enzyme	Control	30 days		180 days		330 days	
		iAs	iAs+BTE	iAs	iAs+BTE	iAs	iAs+BTE
ERCC1	1	0.99±.01	0.99±.01	0.72±.09b	0.96±.07*	0.63±.05^a^	0.90±.03*
ERCC2	1	0.70±.02^b^	0.84±.03**	0.60±.06^a^	0.90±.04*	0.42±.01^a^	0.82±.06*
XRCC1	1	0.60±.03^b^	0.76±.09**	0.46±0.5^a^	0.94±.05*	0.34±.01^a^	0.88±.07*
OGG1	1	1.26±.05^c^	1.14±.02	1.48±.09^a^	1.20±.04*	1.68±.07^a^	1.11±.03*
DNA Ligase I	1	0.43±.04^a^	0.54±.02**	0.28±.08^a^	0.68±.06*	0.13±.06^a^	0.52±.01*
XPA	1	0.74±.09^b^	0.79±.03***	0.42±.02 ^a^	0.64±.03*	0.24±.07^a^	0.51±.03*
PARP1	1	0.62±.01^b^	0.62±.09	0.40±.05 ^a^	0.69±.04*	0.21±.01^a^	0.63±.02*
DNA Ligase IV	1	0.54±.09^a^	0.78±.04*	0.40±.03 ^a^	0.89±.09*	0.28±.03^a^	0.89±.08*
DNA Pkc	1	0.90±.02^c^	0.96±.05	0.78±.03 ^b^	0.90±.06*	0.66±.05^a^	0.85±.08*
Ku70	1	0.72±.04^b^	0.83±.06**	0.58±.03 ^a^	0.94±.05*	0.38±.01^a^	0.78±.07*

Repression of the expression level of enzymes implicated in BER, NER and NHEJ with development of carcinogenesis have been obtained; For OGG1, expression level increases with exposure to iAs and that also with development of carcinogenesis; It is apparent from the band intensities that administration of BTE efficiently modulates the expression level; Significant changes have been observed at 180 and 330 days of exposure to iAs

**Table 3:** Fold Change in the Expression of Cytokines by iAs as Evident from Band Intensities and Its Modulation by BTE

Cytokines	Days	Control	Arsenic	As+Tea
TNF-α	30	1	1.1	1
	180	1	1.45±.03^b^	1.2±.01
	330	1	2.5±.094^a^	1.4±.08*
IL-2	30	1	1	1
	180	1	1.5±.042^b^	1.28±.05***
	330	1	1.7±.045^b^	1.2±.024*
IL-6	30	1	1.2±.012	1.1±.01
	180	1	1.6±.022^b^	1.2±.014**
	330	1	2.3±.072^a^	1.8±.046*
IL-8	30	1	1	1
	180	1	1.5±.013b	1.2±.011***
	330	1	2.2±.014a	1.6±.018**
IL-10	30	1	1	1
	180	1	0.68±.021^b^	0.9±.019**
	330	1	0.46±.01^a^	0.78±.014*
IL-13	30	1	1.1	1.1
	180	1	1.8±.023^a^	1.3±.012**
	330	1	2.3±.034^a^	1.9±.019**
IL-17a	30	1	1	0.96
	180	1	1.9±.06^a^	1.4±.011**
	330	1	2.4±.098^a^	2±.05**
IL-22	30	1	0.98	1
	180	1	1.8±.02^a^	1.28±.03***
	330	1	2.5±.041^a^	1.5±.017*

Alteration of the expression level of cytokines with development of carcinogenesis have been tabulated; Pro-inflammatory cytokine expression increased with the development of skin carcinogenesis, which were repressed by inclusion of BTE; Anti-inflammatory cytokine IL-10 level decreased with exposure time to iAs, but BTE helped to regress the effect; BTE therefore elicits a promising role in iAs induced inflammation; Significant at ^a^ p< 0.0001; ^b^ p< 0.001; ^c^ p< 0.05, with respect to control group; Significant at * p< 0.0001; ** p< 0.001, *** p< 0.5 with respect to iAs group at corresponding exposure time.

**Figure 6:**
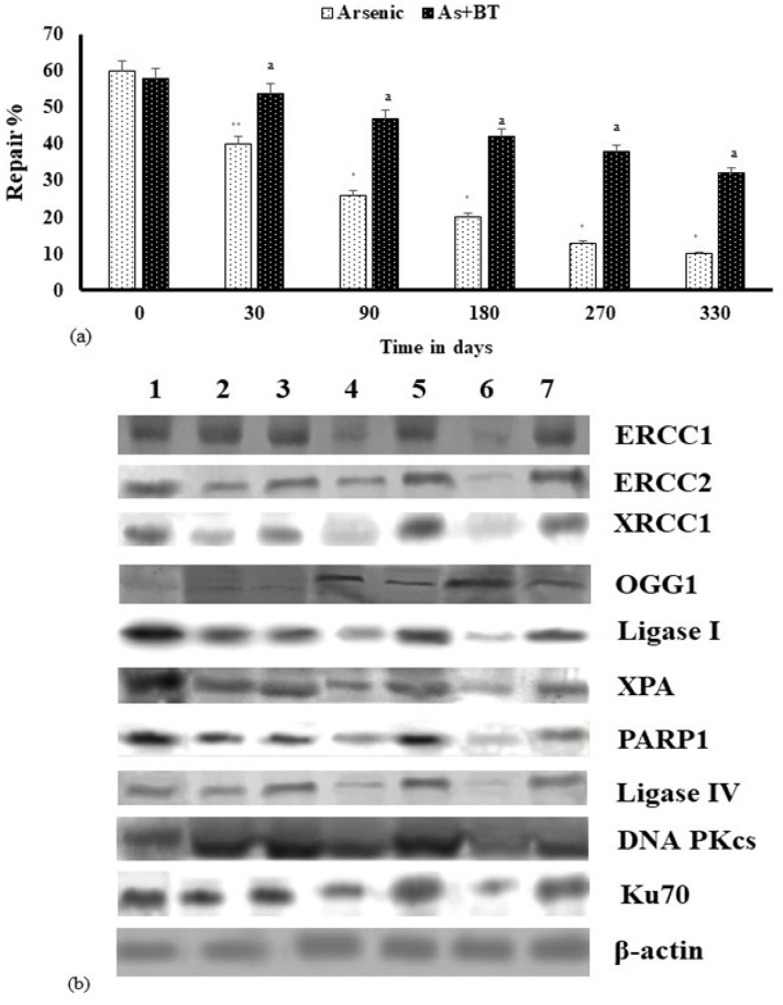
DNA Repair Potential as Affected by iAs. (a) Impairment of repair activity in lymphocytes of mice chronically exposed to iAs and its enhancement by BTE. Reduction in repair activity with respect to control (0 day) is significant at *p< 0.0001; **p<0.001. Elevated repair activity by BTE administration with respect to iAs treated group is significant at ap<0.0001. (b) Expression of BER (namely OGG1, PARP1, XRCC1, DNA ligase I), NER (ERCC 1, ERCC 2, XPA) and NHEJ (DNA PKcs, Ku70 and Ligase IV) at protein level has been represented in Lane 1- Control, Lanes 2, 4, 6 by iAs at 30, 180 and 330 days respectively. Lanes 3, 5, 7 represent the expression as modulated by intervention with BTE at 30, 180 and 330 days respectively. β-actin was used as the loading control

**Figure 7:**
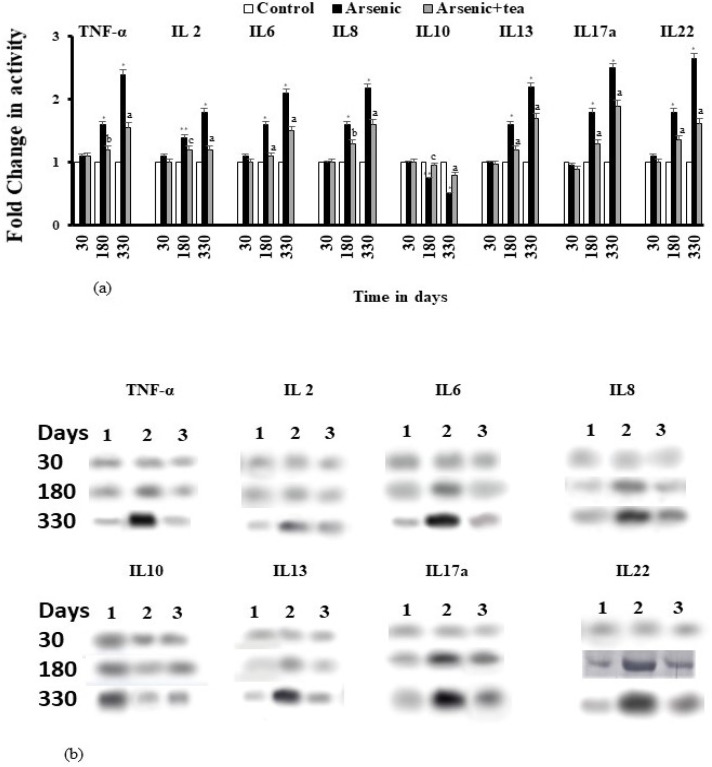
Modulation of Cytokines by iAs during Skin Carcinogenesis, as Modulated by BTE. (a) Levels of cytokines, TNF-α, IL-2, IL-6, IL-8, IL-10, IL-13, IL-17a, IL-22 as assessed by ELISA have been depicted as bar diagram. Values are mean of three independent experiments and are significant at *p<0.0001, with respect to the control value. Modulation of cytokine levels by BTE, with respect to the treated group is significant at ^a^p<0.0001, ^b^p<.001, ^c^p<0.05. (b) Expression levels of TNF-α, IL-2, IL-6, IL-8, IL-10, IL-13, IL-17a, IL-22, as altered by iAs and modulated by BTE have been shown. Lanes 1, 2, 3 signify control, iAs and iAs+BTE treated groups respectively

**Figure 8:**
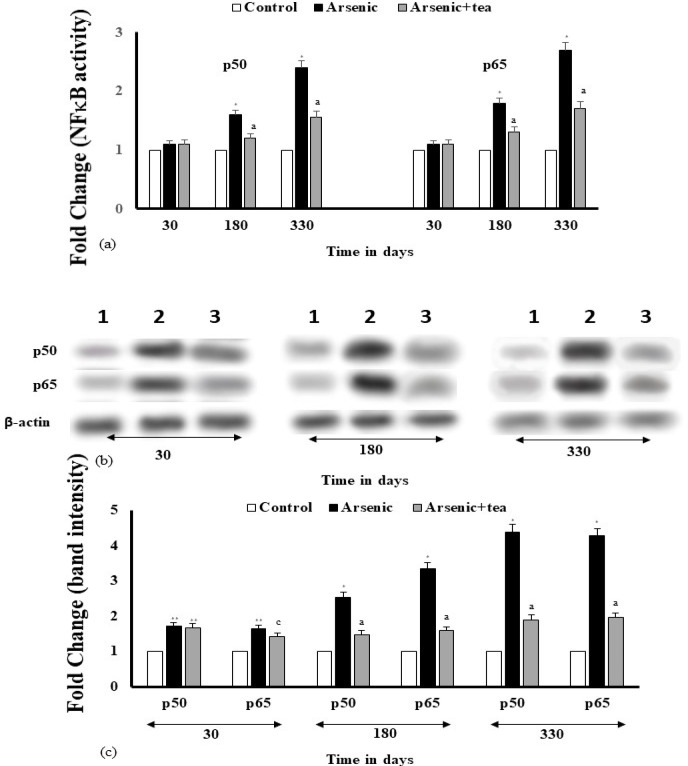
Modulation of NFκB by iAs during skin carcinogenesis, as modulated by BTE. (a) Activity of NFκB as assessed by ELISA, have been depicted as bar diagram. Values are mean of three independent experiments and are significant at *p< 0.0001, with respect to the control value. Modulation of cytokine levels by BTE, with respect to the treated group is significant at ^a^p<0.0001. (b) Expression levels of NFκB, as induced by iAs and modulated by BTE have been shown by Western blot bands. Lanes 1, 2, 3 signify control, iAs and iAs+BTE treated groups. (c) Band intensities (fold change) as obtained from Western blot results have been depicted

## Discussion

Around the globe, exposure to iAs for a long time leads to numerous diseases, rendering iAs to be a major health concern. Though not a classical mutagen, iAs has been listed as a class 1 carcinogen by International Agency for Research on Cancer (IARC) (Klein et al., 2007). The exact mechanism of carcinogenesis by iAs is under investigation, however, generation of free radicals by this metalloid may be responsible for its carcinogenicity (Hu et al., 2020). Present study indicates that excess generation of ROS and RNS by iAs causes damage to DNA, proteins, lipids, impairs DNA repair, alters antioxidant machinery and maintains chronic inflammatory conditions. All these phenomena, alone or cumulatively promote carcinogenesis. Innumerable strategies have been employed for remediation of ground water iAs (Azubuike et al., 2016), which are laborious and expensive, hence cannot be employed on large scale. Countering free radical generation by iAs may be a better alternative. Antioxidants may be a solution for the problem; however, synthetic ones have demerits (Deepmala et al., 2015). Comparatively cheap, naturally available phytochemicals with known mechanisms of action appear to be an accepted solution. Tea being the most popular beverage, may come to the rescue. Green tea is known for its anti-oxidative, anti-inflammatory and anti-cancer potential (Maiti et al., 2019), but people prefer BT over green tea globally. Hence, exploration of BT to control the deleterious effects of iAs may be a novel approach. 

Present study explores the chemo-preventive potential of BT against chronic iAs induced skin carcinogenesis. Histological analysis revealed that after 330 days of treatment with iAs most of the mice developed invasive SCC quite similar to the positive control group of DMBA, elucidating the carcinogenicity of iAs. Co-administration of BTE along with iAs or DMBA resulted in failure of development of both in-situ and invasive SCC in the skin of mice indicating its chemopreventive nature. Excess ROS and RNS generation due to iAs has been found to be reduced in the presence of BTE in Swiss albino mice. DNA damage caused by free radicals, as assessed by SCGE and MN formation has been significantly reduced by BTE. 

The repair system becomes functional when a damage to DNA is detected; if unrepaired, or erroneously repaired, mutation occurs, and its accumulation may lead to cancer. iAs has been found to inhibit repair, by downregulating the enzymes involved in Base excision repair (BER), Nucleotide excision repair (NER) and Non-homologous end-joining (NHEJ). DNA damage and impaired repair function due to iAs may be reversed by BTE, indicating its preventive role in carcinogenesis. 

ROS generation leads to post-translational modifications in proteins by addition of carbonyl moieties like aldehydes, ketones or lactam, besides splitting protein backbone and oxidizing amino acid side chains (Fedorova et al., 2014). Agglomeration of carbonylated species leads to carbonyl-stress, resulting in redundancy of enzymes and increased toxicity. Carbonyl-stress has been linked to numerous other degenerative disorders and inflammation (Fedorova et al., 2014). Results show that BTE dampened the carbonyl-stress, generated due to iAs. Oxidation of poly-unsaturated fatty-acids (PUFA) in the phospholipids of the cell membranes leads to lipid-peroxidation and generation of electrophilic secondary species like malondialdehyde etc. (Fedorova et al., 2014). ROS, reacting with Polyunsaturated fatty acids (PUFA) converts it to a reactive radical, which further reacts with other PUFA molecules, generating more free radicals and lipid-peroxidation products (LPP) (Su et al., 2019). Strong electrophilic LPPs react with nucleophilic amino-acids in the side chains of the proteins and alter their function (Fedorova et al., 2014). The LPPs modify signaling pathways involved in cell death, cell growth etc., leading to promotion of inflammation (Su et al., 2019). Present study shows that BTE represses Lipid-peroxidation in iAs treated mice, another facet of chemo-preventive role of BTE. 

Reduction-oxidation homeostasis is important for maintenance of normal physiological steady state. A regiment of antioxidant enzymes like SOD, catalase, GPx, GR, GST etc. maintain homeostasis by quenching free radicals. Administration of iAs reduces the activity of antioxidant enzymes (Yu et al., 2017), promoting the damage inflicted by ROS; not only DNA-repair is delayed, but many of the repair enzymes involved in repair pathways like BER, NER and NHEJ are down-regulated. This is in agreement with the findings of other research groups (Martinez et al., 2011; Tong et al., 2015; Holcomb et al., 2017). Compromised repair gear results in accumulation of DNA damage, hence mutation, aiding in carcinogenesis. Formation of 8 oxoguanine (8 oxoG), a modified base within DNA, pairing with adenine is triggered by oxidative stress. If 8-Oxoguanine glycosylase (OGG1) fails to repair 8-oxoG through BER pathway, transversion mutation results, which is stable. Oxidative DNA damage is implicated in the development of skin cancers (Kunisada et al., 2005). Increased levels of 8-oxoG and OGG1 have been reported in ulcerative condition, along with prolonged inflammation, due to persistent oxidative stress (Kumagae et al., 2018). The present study shows higher expression of OGG1 in skin cancer tissue due to chronic exposure to iAs. Previous reports from our laboratory revealed that iAs induces 8 oxoguanine (8 oxoG) formation, which resulted in increased OGG1 production. BTE has shown significant reduction in OGG1 expression, exhibiting a promising role in repair.

Chronic, low-level inflammation is intricately associated with cancer (Coussens and Werb, 2002). Oxidative free radicals act as signalling molecules that trigger inflammation; they promote translocation of NF-κB into the nucleus resulting in alteration of cell growth and proliferation. Inflammatory response includes orchestration of numerous pro and anti-inflammatory cytokines which modulate the cellular homeostasis by controlling the transcriptional activity of NF-κB. TNF-α activates NF-κB which in turn alters the apoptotic pathway involving ROS and c-Jun N-terminal kinase (JNK) signalling. Inhibition of ROS suppresses the NF-κB activation as well as IL-6 generation. The pathophysiology of ROS generation and inflammation are thus intertwined and both can regulate each other. This study reveals that iAs maintains chronic inflammatory condition via elevated expression of pro-inflammatory cytokines like TNF-α, IL 2, IL6, IL8, IL13, IL17a and IL22. Two important subunits of the transcription factor NF-κB (p50 and p65) also contribute in this regard. The expression of anti-inflammatory cytokine IL-10 got reduced by iAs. BTE has shown efficacy in countering the inflammatory responses, by up and down-regulating the anti and pro-inflammatory cytokines respectively. 

All the detrimental effects of iAs have been found to be impeded by the goodness of BTE. BTE may thus be considered as a promising candidate in control of skin carcinoma induced by iAs in Swiss albino mice.

## Author Contribution Statement

The experimental work has been executed by Archismaan Ghosh (AG). Animal experimental part was guided by Sutapa Mukherjee (SM). Madhumita Roy (MR) is responsible for designing of experiments, overall supervision and analysis of data. MR and AG prepared the manuscript. 

## Funding Statement

The authors are indebted to Director, Chittaranjan National Cancer Institute (CNCI), Kolkata for providing financial support from institutional intramural fund. AG is indebted to CNCI for his fellowship. Authors are also indebted to Director, Chittaranjan National Cancer Institute (CNCI) for infrastructural facilities. 

## Part of Student’s Thesis

The work is a part of the PhD thesis work of Archismaan Ghosh. The study has been approved by the Department of Life Sciences and Biotechnology, Jadavpur University as a part of his Ph.D thesis work.

## Ethical Issues

The work is based on animal model. The project has been approved by the institutional animal ethical committee (IAEC 1774/MR-3/2017/9) before commencement of study. 

## Conflicts of interest

There has been no conflict of interest among authors. 
